# Apolipoprotein E Genotype in Very Preterm Neonates with Intrauterine Growth Restriction: An Analysis of the German Neonatal Network Cohort

**DOI:** 10.1155/2017/2837027

**Published:** 2017-04-05

**Authors:** Stephen Norda, Tanja K. Rausch, Thorsten Orlikowsky, Matthias Hütten, Sören Schulz, Wolfgang Göpel, Ulrich Pecks

**Affiliations:** ^1^Department of Obstetrics and Gynecology, University Hospital of the RWTH Aachen, Aachen, Germany; ^2^Department of Medical Biometrics and Statistics, University of Lübeck, Lübeck, Germany; ^3^Department of Neonatology, University Children's Hospital of the RWTH Aachen, Aachen, Germany; ^4^Department of Pediatrics, University Hospital UKSH Lübeck, Lübeck, Germany; ^5^Department of Obstetrics and Gynecology, University Hospital UKSH Kiel, Kiel, Germany

## Abstract

*Aim.* Cord blood of intrauterine growth restricted (IUGR) neonates displays lipid changes towards atherosclerotic profiles. Apolipoprotein E (ApoE) and its isoforms (e2, e3, and e4) are involved in the regulation of lipid metabolism. Specifically, ApoE e4 has been associated with atherosclerotic diseases, while e2 has a favorable effect. We therefore hypothesized that ApoE e4 haplotype is frequently observed in IUGR neonates and contributes to impaired fetal growth and the association of IUGR with cardiovascular and metabolic diseases later in life.* Methods.* A cohort of 4885 preterm infants (≥22+0 and <32+0 weeks of gestation and birth weight below 1500 g) from the GNN study cohort was analyzed. Neonates were categorized into subgroups of <3rd, 3rd–10th, and >10th birth weight percentile. Analysis of the single nucleotides rs429358 and rs7412, identifying the ApoE genotype, was carried out using TaqMan® SNP genotyping assays. The proportional odds model was used to assess data.* Results.* No association was found between genotype and birth weight percentiles in each of the subgroups.* Conclusion.* ApoE genotype and low birth weight depict two distinct risk factors for cardiovascular disease without being directly associated.

## 1. Introduction

Inadequate birth weight, compared to population based standards, has a complex pathophysiology not yet entirely understood. Disrupted placentation and consequently insufficient placental vascular supply of nutrients and oxygen to the fetus, among other factors, are believed to be involved in intrauterine growth restriction (IUGR) and to result in small for gestational age (SGA) neonates [1, 2]. As such it shares common pathomechanisms with preeclampsia. Moreover, preeclampsia and IUGR often occur simultaneously especially if the onset is early preterm [3, 4]. Hence, both diseases have been described as “placental syndrome.” The clinical outcome of IUGR born babies is worse compared to neonates born with normal weight. Restricted fetal growth is believed to increase the risk for adverse neurophysiologic development, cardiovascular disease, and dyslipidemia in later life [5–9]. Atherogenic fetal serum lipid configurations have been associated with IUGR by previous research. IUGR born babies were found to have lower concentrations of high-density lipoprotein cholesterol (HDL-C), known for having an anti-inflammatory effect and protective properties against the development of atherosclerosis, while triglycerides and oxidized low-density lipoprotein (oxLDL) levels were elevated in samples of umbilical blood compared to adequate weight newborns [5, 10, 11]. The results propose that disrupted cholesterol and triglyceride handling plays a role in causing suboptimal fetal development and exposes the newborn to an atherosclerotic environment early, consequentially giving raise to irreversible damage to vessels [6].

Apolipoprotein E (ApoE) is an important circulating serum protein involved in transporting lipids and cholesterol and regulating lipid levels. Its regulatory functions have been attributed to many biophysiological processes including neuronal growth and modulation of oxidant and inflammatory processes [12, 13]. The ApoE gene has three allelic variants (e2, e3, and e4). The three haplotypes are determined by the SNPs rs429358 and rs7412. Variations in these nucleotides are determined by nucleobases T-T (e2), T-C (e3), and C-C (e4) [14, 15]. Despite their minor structural disparities, the three variants e2, e3, and e4 and their corresponding amino acid polymorphisms exhibit differences in individual binding properties to different receptors and display diverse effects on lipoprotein metabolism [12].

The association of ApoE e4 with the development of dyslipidemia and cardiovascular disease has been described for the past two decades and is now well established [16, 17]. Recent studies provided evidence of isoform e4 being present in individuals afflicted with severe cerebral palsy, promoting the development of Alzheimer's disease, and promoting the development of epilepsy. On the contrary, possibly due to its diverse binding properties, isoform e2 displays a protective effect [13, 18, 19]. In newborns, carrying the ApoE e2 allele has been associated with lower fetal cord blood LDL-C levels and higher levels of HDL-C suggesting a beneficial effect of this genotype on blood lipid configuration [20, 21].

Given the significant impact of ApoE genotype on serum lipid levels and the association of IUGR with altered lipid metabolism, we hypothesized fetal ApoE genotype to be a modulator of fetal growth and severity of IUGR. A link between ApoE genotype and birth weight percentile would prove the role of the APOE gene as modulator of fetal growth and consequently provide an explanation for the fact that impaired fetal development depicts a cardiovascular risk factor. Hence, the aim of the present study was to identify ApoE genotypes in IUGR neonates. We took advantage of a nation-wide genomic study of the German Neonatal Network (GNN) including more than 18000 preterm neonates born before 32 weeks of gestation or with very low birth weight below 1500 g. The neonates were clustered according to birth weight percentiles with the lowest percentiles likely reflecting impaired fetal development. However, IUGR is not uniquely defined and neonates can be born small for their gestational age (SGA) by genetic determination and in the absence of a pathologic process [5, 22]. Hence, in this study different SGA subtypes and scenarios have been considered including different cut-offs of percentiles to define SGA as well as factors associated with IUGR like hypertensive disorders in pregnancy. Identifying ApoE genotypes as an individual risk factor of IUGR or severity of IUGR has the potential to allow for better individual prenatal observation and postnatal treatment especially in preterm born neonates in need of intensive neonatal care.

## 2. Methods

### 2.1. Study Cohort

Subjects were enrolled in the GNN cohort between January 2009 and December 2015 by 54 currently participating neonatal intensive care units in Germany. Preterm infants with a birth weight below 1500 g and born <37+0 weeks of gestation were enrolled. The GNN study was approved by the local committee for research in human subjects of the University of Lübeck (Germany), as well as by all of the local committees of participating centers. Written informed consent was obtained from parents with neonates eligible for the research and publication of data. A predefined clinical data set form, containing obstetric and neonatal data, was filled out by the attending physician. Data were then sent to the GNN coordinating center in Lübeck. Parental refusal, early neonatal death, and parents not asked to participate were reasons for nonenrollment. A specialized neonatologist surveyed the data quality by annual visits to the participating centers. Obstetrical and neonatal data collected included maternal origin, maternal age, gestational age, birth weight, fetal sex, singleton versus multiple birth, fetal malformations, presence of pathologic Doppler/IUGR, presence of maternal pregnancy induced hypertension (PIH) or preeclampsia, and presence of HELLP syndrome. Birth weight percentiles were calculated according to Voigt et al. [23]. Ultrasound measurements executed between 8 and 12 weeks of gestation determined fetal age.

### 2.2. Genotyping

ApoE genotyping was performed on buccal swabs and/or cord tissue transferred to the study center (University of Lübeck) together with the clinical data set form. DNA extraction was carried out by using commercial DNA purification kits (Qiagen, Hilden, Germany). The samples underwent real-time PCR. Genotyping of the APOE rs429358 and rs7412 single nucleotide polymorphisms (SNP), defining the e2, e3, and e4 allele, was performed by using TaqMan SNP Genotyping Assays (Applied Biosystems, Foster City, USA) according to the manufacturer's protocol. Genotyping of rs7412 and rs429358 was done in 10211 GNN-infants who were born between 2009 and 2014. Genotyping was successful in 93% of all patients for rs429358 and 96% of all patients for rs7412 (90% for both SNPs).

### 2.3. Patient Selection

**Figure 1:**
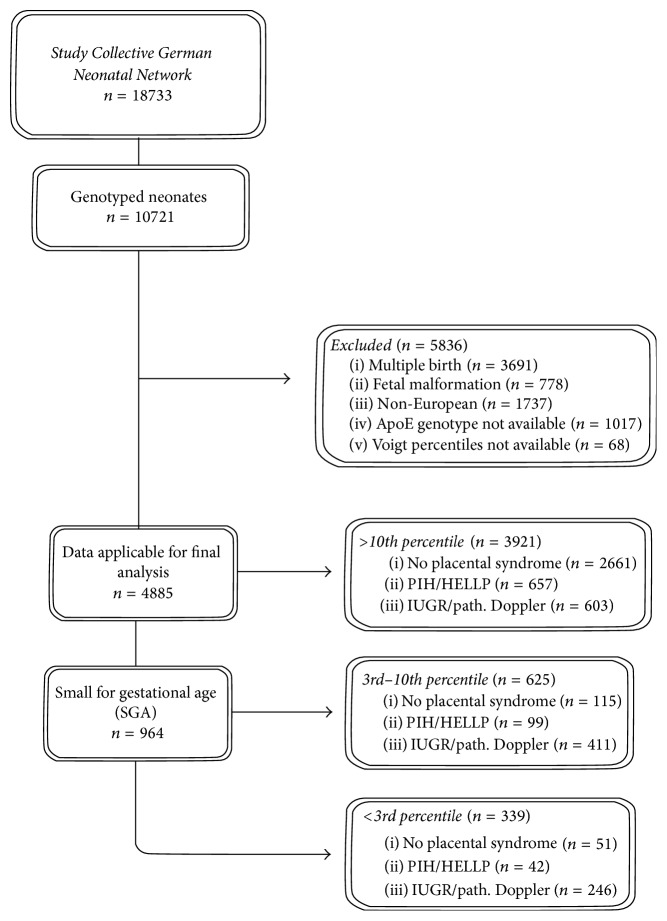
Inclusion/exclusion algorithm of probands of the German Neonatal Network. Multiple reasons for exclusion could apply. PIH includes preeclampsia. Patients with both “PIH/HELLP” and “IUGR/pathological Doppler ultrasound” were grouped into the “IUGR/pathological Doppler ultrasound” subgroup.

**Figure 2:**
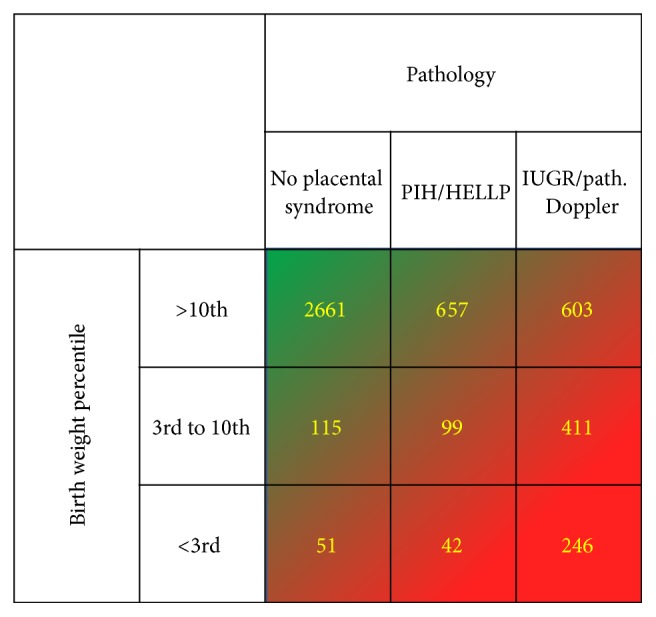
Frequencies of birth weight percentile groups and the presence or absence of PIH/HELLP syndrome or IUGR/pathological Doppler ultrasound as clinically indicated, of the total 4885 preterm neonates included into the analysis. Patients with both “PIH/HELLP” and “IUGR/pathological Doppler ultrasound” were grouped into the “IUGR/pathological Doppler ultrasound” subgroup.

We selected preterm infants with gestational age below 32+0 weeks, a birth weight below 1500 grams, and European origin. Infants from multiple birth and with fetal malformations were excluded (Figure 1). To estimate interactions between ApoE genotype and abnormal fetal growth different scenarios were established: 4885 preterm neonates eligible for analysis were divided into three birth weight percentile groups: >10th percentile (*n* = 3921), 3rd–10th percentile (*n* = 625), and <3rd percentile (*n* = 339). Those groups were further divided into subgroups according to antenatal clinical diagnosis of one of the subsequent features indicating a placental involvement (placental syndrome): (1) presence of maternal pregnancy induced hypertension (PIH including preeclampsia) and/or HELLP syndrome, (2) presence of IUGR and/or pathologic Doppler (maternal and/or fetal side) with/without PIH/HELLP, or (3) absence of criteria (1) or (2) (no placental syndrome). It was assumed that the subgroup with birth weight percentile <3rd plus the clinical indication of IUGR/pathologic Doppler was most severely growth restricted while the subgroup of patients with birth weight percentile >10th in the absence of (1) or (2) served as the most “pure” control group (Figure 2).

### 2.4. Statistical Analysis

Data analysis was performed using the SPSS 20.0 data analysis package (IBM, Munich, Germany). In order to evaluate the effect of the ApoE genotype on the birth weight in different SGA subtypes the proportional odds model was used to assess ordinal data. The model analyzes the likelihood to reach a higher ordinal level in relation to other variables. An equal slopes assumption of the data must be met which is tested beforehand. Our null hypothesis was (*H*_o_) = the ApoE genotype not affecting the Voigt birth weight percentile in different subgroups of premature IUGR. The type I error level was set to 0.05. To correct for the multiple testing, nominal *p* values were adjusted according to the Bonferroni-Holm procedure. Hardy Weinberg equilibrium for three alleles was assessed by chi-square test.

## 3. Results

Maternal and neonatal patient characteristics for each subgroup are shown in Table 1. The average maternal age was 30.19 years (SD ± 5.92). Gestational age on average was slightly smaller in the >10th percentile weight group (28,28 ± 2.33 weeks) than in the <3rd percentile weight group (29.92 ± 4.25 weeks). Mean birth weight of >10 percentile group was 1075 g (SD ± 275 g), 924 g (SD ± 347 g) in the 10th–3rd, and 839 g (SD ± 421) in the <3rd group. The percentage of female neonates varied from 47.4% to 50.4% among subgroups. From the total group of 4885 probands, close to 95% of values were available for analysis of maternal and neonatal patient characteristics.

The frequencies of birth weight percentiles of neonates born without placental syndrome, in the presence of PIH/HELLP or IUGR/pathological Doppler, respectively, are displayed in Figure 2.

Overall, haplotype frequencies were e2: 7,4%, e3: 79%, and e4: 13,6%, respectively. ApoE genotype combinations found in our study population are shown in Table 2. The genotypes showed a normal distributed Hardy Weinberg equilibrium. Genotype distribution per birth weight percentile and functional subgroup is depicted in Table 3.

Using proportional odds model and cumulative link model, respectively, interactive influence of ApoE genotypes and placental syndromes (three categories) on birth weight centiles have been estimated. As expected the groups PIH/HELLP and IUGR/pathological Doppler showed a statistically significant negative effect on birth weight percentile when compared to neonates born in absence of a placental syndrome (Table 4). None of the neonatal ApoE genotypes could be identified as having an influence on birth weight percentiles when e2/e2 was used as a reference group.

To further investigate a possible correlation between ApoE genotype and birth weight percentile, the ApoE genotypes were pooled in groups containing the haplotype e2 (e2/e2 and e2/e3), haplotype e3 only, and haplotype e4 (e3/e4 and e4/e4). Thus the compiled allele groups did not show a significant effect on the birth weight percentiles in any of the analyzed subgroups compared to the groups e2/e2 and e2/e3 (data not shown).

## 4. Discussion

To our knowledge, the data set used in this study was the largest analyzed to date. 4885 preterm neonates were included in the analysis with the aim of identifying the effect of the ApoE genotype on proper fetal development. However, we did not find any association of ApoE haplotypes with birth weight percentiles independently of whether or not pregnancy was further complicated by a placental disorder characterized by PIH or IUGR.

Three prior studies investigated the ApoE genotype in IUGR/low birth weight children. Our results are in accordance with Akisu et al. who also found no correlation between the ApoE genotype and IUGR born newborns [24]. However, their results are not entirely applicable to the present study since all infants investigated had completed the 36th week of gestation. Moreover, the study was massively underpowered as it included only 20 cases of IUGR. Another study by Szitanyi et al. retrospectively investigated the ApoE genotypes and birth weight of two groups of 10-11-year-old children. They hypothesized that both intrauterine undernutrition, demonstrated by a lower birth weight, and the ApoE genotype participate in the development of hypercholesterolemia in childhood [25]. Children were subdivided into tertiles with high and low levels of cholesterol, respectively. While the high-cholesterol group had significantly lower birth weight (0.3 kg) and ApoE e4 had a higher prevalence among this group, no association could be established between ApoE e4 and birth weight alone. Unfortunately, the study did not provide any data on weeks of gestation at birth, actual birth weight, birth weight percentiles, or the presence of IUGR/SGA in their study population of 139 children. Therefore, their results need to be addressed with caution. A third large study by Infante-Rivard et al. (449 newborns) using a family based study design consistently found a significantly reduced transmission of allele e2 to newborns affected with IUGR defined as birth weight below the 10th percentile. The authors concluded that allele e2 is protective against IUGR. However, their diagnosis of IUGR needs to be questioned, since birth weight percentile below the 10th percentile alone is not a reliable definition of IUGR. The authors themselves stated that exclusion of patients with histopathologically confirmed placental infarctions (*n* = 10) results into an even more statistically significant effect. This points to different pathomechanisms for specific IUGR/SGA subgroups [20].

Though we did not primarily aim to analyze the association between ApoE genotype and the incidence of preeclampsia and the HELLP syndrome, our study does partly allow us to draw certain conclusions. Both of the aforementioned diseases are pathologically closely related to IUGR. No association between the clinical diagnosis of pregnancy being terminated in the presence of a hypertensive disorder like preeclampsia or the HELLP syndrome has been observed in this study. The present study therefore contradicts a recent publication by Procopciuc et al. in which the fetal Apo e4 genotype was found to be an independent risk factor for preeclampsia compared to the other ApoE genotypes [26]. The diverging conclusions of their study are probably caused by the small sample-size (*n* = 141 with 47 patients suffering on preeclampsia) with ApoE e4 not being normally distributed in their control group. Other authors did not find associations of ApoE genotype and the risk of preeclampsia when genotyping the mother [27–29] which corresponds with our observations in the fetus.

Despite these findings, there is increasing evidence that ApoE plays a pivotal role in fetal development and lipid metabolism. ApoE is the only apolipoprotein in the fetal circulation in concentrations as high as the mother's [30]. It is highly associated with fetal HDL-C that, in contrast to adults, is the predominant lipoprotein in the fetus at term [31]. In cord blood of fetuses with IUGR, both HDL-C and ApoE concentrations are largely reduced [32]. Interestingly, maternal ApoE genotype impacts fetal lipid concentration levels and vice versa. While the e2 isoform in newborns is associated with elevated maternal LDL-C and apolipoprotein B (ApoB) levels, the same isoform in the mother is associated with lower LDL-C and ApoB levels and higher HDL-C levels in cord blood [21, 33]. Maternal cholesterol in humans rises during pregnancy to guarantee fetal nutritional supply. Furthermore, cholesterol levels are affected by a number of factors, environmental and genetic [34]. One influencing candidate on cholesterol levels is therefore the* APOE *locus, either through its direct effects on the developing fetus, on the fetal lipoprotein metabolism, or through its effects on maternal cholesterol levels and the maternofetal lipoprotein metabolism. In IUGR fetal serum lipid levels resemble atherogenic profiles, with lower HDL-C concentrations and higher triglyceride levels as well as elevated ratios of oxidized LDL/LDL-C and LDL-C/HDL-C [5, 10, 11]. The relationship is believed to derive from the early susceptibility of tissue to damage while being in a state of plasticity [6, 7].

The physiologic effect of the ApoE genotype on lipoprotein levels and the development of atherosclerotic diseases are well established in humans. Though being born with low birth weight predisposes to cardiovascular disease later in life it is likely that the three haplotypes of the APOE gene and low birth weight depict two independent risk factors on cardiovascular disease, since our study does not suggest that the association between low birth weight and cardiovascular disease is due to ApoE genotype.

Our study is limited by two main aspects. First, preterm birth per se is a pathological condition; hence, our study lacks a valid control group of uncomplicated pregnancies. However, the overall distribution of ApoE genotypes was within the expected range for the German population. Second, the study may be biased due to the vague definition of IUGR in the absence of precise antenatal recorded criteria. Neonates with birth weights less than the 10th percentile of a population are SGA. The term SGA is descriptive and refers not to fetal growth velocity but to the birth weight of an infant. It does not reflect the causes that lead to the low birth weight. Most SGA neonates are born small by constitution and have constant growth patterns [5, 22, 35]. By contrast, IUGR indicates the presence of a pathophysiological process that slows down or inhibits fetal growth at a certain but usually unknown developmental stage during pregnancy. Hence, the diagnosis of IUGR is based ideally on the antenatal observation of a deceleration of fetal growth velocity by serial sonographic measurements showing a “crossing of percentiles” or other antenatal parameters of fetal well-being [22, 36]. Evidence is increasing that IUGR is a heterogeneous disorder that can be subdivided into clinical presentation of concomitant preeclampsia and early onset or late onset IUGR with a cut-off at 32 to 34 weeks of gestation [2, 37]. Both, preeclampsia and increased resistance of the umbilical arteries (as indicated by resistance index/pulsatility index in Doppler sonography) are often associated with severe early onset IUGR. The indication of the presence of PIH, HELLP syndrome, IUGR, and antenatal pathological Doppler parameters in this study relies on rather clinical documentations that has not followed a strict study protocol and is not uniquely defined within the participating centers, since this was not the primary study aim of the GNN study. However, the inclusion criteria in this study are restricted to neonates born very preterm (early onset) making it a rather homogeneous study population. Moreover, the large size of our study population allowed us to investigate subgroups of very-low-birth-weight-infants according to various definitions of IUGR with high accuracy and low chance of error.

## 5. Conclusion

Genotyping our large cohort of 4885 preterm infants did not reveal an effect or dose-effect relationship of the fetal ApoE isoform on the birth weight percentile in all subgroups investigated. IUGR born infants display atherogenic blood lipid levels. Apolipoprotein E is known to regulate blood lipids and its three haplotypes are associated with distinct blood lipid patterns. Yet, the hypothesis of ApoE genotype affecting birth weight could be rejected since no direct effect was found. The stratification of maternal pathology in our large population size, that in earlier studies might have biased the results, could not alter this conclusion. Cholesterol levels and ApoE genotype depict major risk factors for CVD in later life. Our results, however, suggest that the ApoE genotype and low birth weight represent two synergistic factors exposing the fetus to an atherogenic environment and contributing to intrauterine programming without being directly associated to each other. The maternal genotype, known for affecting fetal blood lipids, could play a role and should be addressed in further studies.

## Figures and Tables

**Table 1:** Patient characteristics. Displayed values are based on data available.

	>10P		3–10P		<3P	
	Mean	(SD)	Mean	(SD)	Mean	(SD)
Maternal age (years)	30.20	(5.90)	30.04	(5.94)	30.36	(6.05)
Gestational age at birth (weeks)	28.28	(2.33)	29.74	(3.13)	29.92	(4.25)
Birth weight (gram)	1075	(275)	924	(347)	839	(421)

	frequency	(%)	frequency	(%)	frequency	(%)

Neonatal gender (*n*, % female)	1783	(47.4)	593	(50.4)	325	(48.3)

**Table 2:** Genotyping of the 4885 neonates.

Genotype	e2/e2	e3/e2	e3/e3	e3/e4	e2/e4	e4/e4
*n*	28	569	3049	1050	94	95
%	0.54	11.63	62.39	21.57	2.01	1.86

**Table 3:** Genotype distribution (frequencies) of preterm neonates subgrouped by birth weight percentile and type of pregnancy related pathology. Patients with both, “PIH/HELLP” and “IUGR/pathological Doppler ultrasound,” were grouped into the “IUGR/pathological Doppler ultrasound” subgroup.

Placental syndrome	Birth weight percentile	e2/e2	e3/e2	e3/e3	e3/e4	e2/e4	e4/e4
No placental syndrome	>10	15	321	1644	572	57	52
	3–10	1	17	70	22	3	2
	<3	1	8	28	12	1	1

PIH/HELLP	>10	7	72	417	135	13	13
	3–10	0	10	61	26	1	1
	<3	0	2	29	9	0	2

IUGR/path. Doppler	>10	1	63	399	117	9	14
	3–10	0	52	243	105	5	6
	<3	3	24	158	52	5	4

**Table 4:** Analysis of interactive effect of ApoE genotype and subgroups with placental syndrome (PIH/HELLP, IUGR/pathologic Doppler) on birth weight percentile groups.

Factor	Estimate	SE	*z* value	*Nominal P*	*Adjusted P*
e2/e3	0.4891	0.5359	0.9127	0.36	1
e3/e3	0.5667	0.5256	1.0781	0.28	1
e3/e4	0.4305	0.5297	0.8128	0.42	1
e4/e4	0.7221	0.6048	1.1939	0.23	1
e2/e4	0.5635	0.6080	0.9268	0.35	1
PIH/HELLP	−1.2365	0.1224	−10.1034	<0.0001	<0.0001
IUGR	−2.8398	0.0971	−29.2359	<0.0001	<0.0001

Results of analyzing the effect of individual ApoE genotypes with underlying maternal pathology, respectively, on birth weight percentile. Data were compared to reference group e2/e2 for genotype analysis and to no placental syndrome, respectively.

Patients with both, “PIH/HELLP” and “IUGR/pathological Doppler ultrasound,” were grouped into the “IUGR/pathological Doppler ultrasound” subgroup. All values are rounded on four decimals. The type I error level was set to 0.05. Adjusted *p* values are adjusted according to the Bonferroni-Holm procedure.
